# Evaluating the Role of Aspirin in Liver Disease: Efficacy, Safety, Potential Benefits and Risks

**DOI:** 10.3390/life14121701

**Published:** 2024-12-23

**Authors:** Amani Elshaer, Blanca C. Lizaola-Mayo

**Affiliations:** 1 Department of Internal Medicine, Mayo Clinic, Phoenix, AZ 85054, USA; elshaer.amany@mayo.edu; 2 Division of Gastroenterology, Hepatology and Transplant Hepatology, Mayo Clinic, Phoenix, AZ 85054, USA

**Keywords:** aspirin, chronic liver disease, hepatocellular carcinoma, metabolic-associated steatotic liver disease

## Abstract

The rise in liver disease incidence and prevalence has led to increasing morbidity and mortality worldwide. Persistent hepatic inflammation drives disease progression by increasing fibrosis, advancing to cirrhosis, and potentially developing into hepatocellular carcinoma (HCC). Addressing these complications is essential to reduce liver-related mortality. Recent studies suggest that non-steroidal anti-inflammatory drugs, particularly aspirin, may play a beneficial role in managing liver disease. Aspirin’s anti-inflammatory and chemoprotective effects contribute to slowing disease progression and reducing the risks associated with chronic liver disease (CLD). This review highlights the current literature on the effects of aspirin in CLD, with a focus on patients with metabolic-associated steatotic liver disease (MASLD) and hepatitis B and C. We will examine aspirin’s potential ability to mitigate fibrosis, reduce the incidence of HCC, and lower liver-related mortality. Additionally, we will discuss its potential side effects and safety considerations, particularly in the context of liver disease, where there is an increased risk of bleeding.

## 1. Introduction

The incidence and prevalence of liver disease are rising globally, resulting in significant morbidity and mortality [1]. Chronic liver disease (CLD) can stem from various etiologies, including viral hepatitis, metabolic-associated steatotic liver disease (MASLD), alcohol-related liver disease, autoimmune conditions, primary biliary cholangitis, among others. Persistent inflammation is a central driver of disease progression, leading to fibrosis and eventually cirrhosis, which can be further complicated by the development of hepatocellular carcinoma (HCC). HCC accounts for 3.5% of all deaths worldwide [2]. According to the Global Burden of Diseases, it estimates that approximately 2.1 million deaths occur each year due to CLD, with 62% of these attributed to cirrhosis and 38% to HCC [1,3]. This highlights the severity of liver diseases and emphasizes the critical need to mitigate its risk factors and reduce its impact.

The progression of liver disease is primarily driven by persistent inflammation. As a result, various treatments—including antioxidants, lipid-lowering drugs, antiplatelet agents, and antidiabetic medications—were explored in the management of CLD [4]. This study will specifically focus on aspirin and its anti-inflammatory properties in mitigating these risks in CLD. By reducing inflammation, aspirin may help slow the progression to fibrosis [5]. Furthermore, aspirin exhibits promising chemopreventive effects, which could potentially lower the incidence of HCC [6]. Thus, this study aims to summarize the key aspects of aspirin’s use in managing liver disease, offering a comprehensive overview of its efficacy and safety in this context.

## 2. Aspirin’s Mechanism of Action

**Figure 1:**
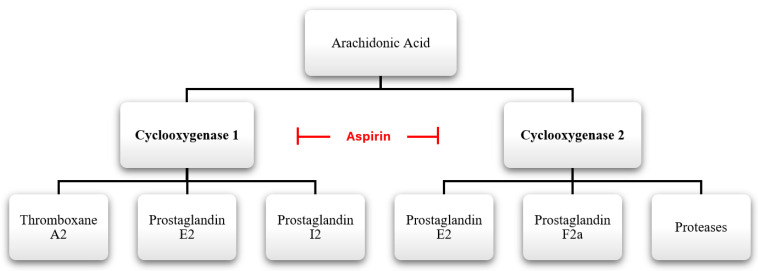
Illustration of aspirin’s mechanism of action.

Aspirin works by irreversibly inhibiting cyclooxygenase 1 (COX-1) and cyclooxygenase 2 (COX-2) enzymes, as illustrated in Figure 1 [7,8]. By inhibiting the COX-1 enzyme, aspirin prevents the conversion of arachidonic acid to prostaglandin H2 (PGH2) and thromboxane A2 (TXA2) [7,8]. Through this inhibition, the use of aspirin will lead to decreased platelet activation, aggregation, and vasoconstriction; thus, contributing to its antithrombotic effect [7,8]. However, by inhibiting COX-1, aspirin also reduces the production of cytoprotective prostaglandins such as prostaglandin I2 in the gastrointestinal (GI) tract. This may lead to GI complications such as gastritis, peptic ulcers, and bleeding [9,10]. Additionally, COX-1 plays a role in maintaining renal blood flow and glomerular filtration rate, which may become compromised with COX-1 inhibition [11].

COX-2 plays a crucial role in inflammation through several mechanisms. It increases NF-κB activation, which upregulates the expression of pro-inflammatory cytokine interleukin-1β (IL-1β) and NLRP3 (NOD-, LRR-, and pyrin domain-containing protein 3), both of which are critical pro-inflammatory cytokines and potent mediators of inflammation [12]. Therefore, by inhibiting the COX-2 enzyme, aspirin prevents the production of pro-inflammatory prostaglandins, including prostaglandin E2 (PGE2), prostaglandin D2 (PGD2), prostaglandin F2α (PGF2α), and prostacyclin. Instead, aspirin promotes the formation of 15(R)-hydroxyeicosatetraenoic acid (15(R)-HETE) [7,13]. 15(R)-HETE has been shown to inhibit platelet aggregation and modulate inflammation, further contributing to aspirin’s therapeutic effects [13,14].

Aspirin also has chemopreventive properties, primarily by mitigating inflammation and influencing cancer-related pathways. The COX-2 enzyme plays a key role in both inflammation and cancer progression by producing PGE2 [15]. Elevated PGE2 levels are known to stimulate cancer cell proliferation and inhibit apoptosis. Additionally, COX-2 facilitates angiogenesis by producing vascular endothelial growth factor (VEGF), which is essential for tumor growth and metastasis. By blocking COX-2, aspirin reduces PGE2 levels thereby decreasing its tumorigenic effects. Furthermore, the inhibition of PGE2 leads to the suppression of the Wnt/β-catenin pathway, which is a significant driver in the development of colorectal cancer [16,17].

## 3. Aspirin’s Mechanism of Action in the Liver

To thoroughly understand the effects of aspirin on liver function, it is essential to examine the role of platelets in liver inflammation. Research indicates that platelets are pivotal in driving inflammation and advancing liver disease through various mechanisms. A study by Malehmir et al. demonstrated that platelet counts are significantly elevated in patients with metabolic-associated steatohepatitis (MASH) compared to those with simple steatosis, highlighting a correlation between platelet levels and inflammation [18]. The platelets adhere to the liver vasculature using platelet glycoprotein Ibα (GPIα) and mediate interactions with Kupffer cells and extracellular matrix [18]. This interaction promotes the recruitment of immune cells to the liver and stimulates the release of cytokines and chemokines, which exacerbate the inflammatory process. The sustained release of these pro-inflammatory mediators leads to the activation of hepatic stellate cells and the recruitment of neutrophils and other immune cells, ultimately contributing to the development of fibrosis [18]. Thus, aspirin acts by inhibiting platelet function thereby reducing the associated inflammation.

**Figure 2:**
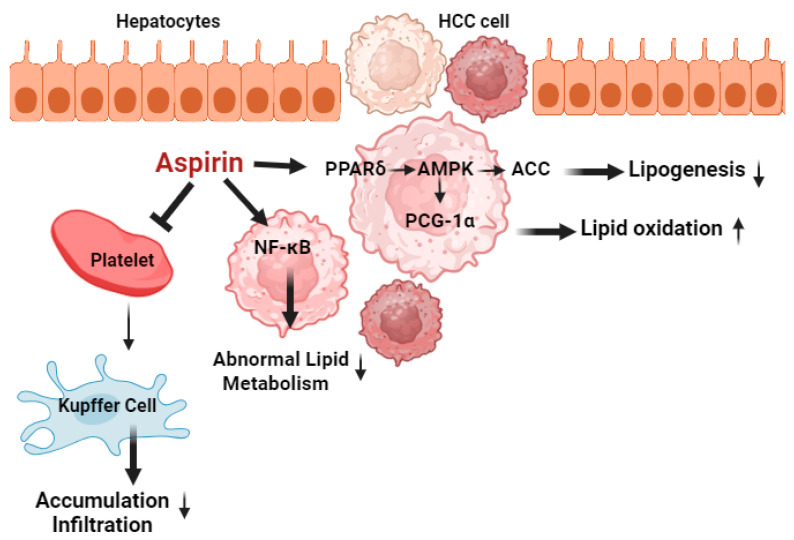
Aspirin’s mechanism of action in liver cells.

Another critical aspect of CLD and HCC development is obesity as it facilitates the activation of the janus kinase pathway, resulting in elevated levels of leptin, as well as pro-inflammatory, pro-angiogenic, and pro-fibrogenic cytokines. This process ultimately leads to increased lipid accumulation leading to lipotoxicity, elevated reactive oxygen species, and endoplasmic reticulum stress [19]. Ongoing inflammation will then progress from simple steatosis to steatohepatitis, fibrosis, and cirrhosis. Cirrhosis would significantly increase the risk of HCC development [19]. Aspirin helps in reducing the levels of hepatic lipid accumulation and inflammation through upregulating the levels of peroxisome proliferator-activated receptor delta (PPARδ), AMP-activated protein kinase (AMPK), and peroxisome proliferator-activated receptor gamma coactivator 1-alpha (PGC-1α) in liver cells [20]. The presence of these proteins helps in lipid metabolism and oxidative phosphorylation though different mechanisms. PPARδ enhances fatty acid oxidation through autophagy lysosomal pathways, which reduces the intrahepatic lipid content [21]. AMPK activation downregulates the lipogenic genes, leading to reduced fatty acid and cholesterol synthesis [22]. The PGC-1α pathway increases the expression for genes involved in fatty acid oxidation [23]. Therefore, by enhancing the levels of these proteins, the hepatic lipid levels will be reduced (Figure 2).

Aspirin affects the peroxisome proliferator-activated receptor delta (PPARδ) and activates AMP-activated protein kinase (AMPK), leading to the phosphorylation and subsequent inhibition of Acetyl-CoA Carboxylase (ACC). ACC is a crucial enzyme in fatty acid synthesis, and its inhibition leads to decreased lipid synthesis and enhanced fatty acid oxidation. Additionally, aspirin downregulates the nuclear levels of Nuclear Factor Kappa B (NF-κB), reducing lipogenesis, triglyceride, and cholesterol levels in hepatocellular carcinoma (HCC) cells. By inhibiting the NF-κB pathway, aspirin is also able to inhibit Kupffer cell activity thereby reducing pro-inflammatory cytokine production, hepatic inflammation, and fibrosis. Furthermore, aspirin inhibits platelet aggregation by irreversibly acetylating COX-1, which decreases TXA2 production and promotes platelet apoptosis.

Given aspirin’s noted chemopreventive effects, ongoing research is investigating its potential role in preventing HCC. A study by Hossain et al. examined aspirin’s effects on HCC cells both in vitro and in vivo. The study found that aspirin effectively inhibited the growth of HepG2 human HCC cells and induced apoptosis through both extrinsic and intrinsic pathways, as is evidenced by alterations in the Bax/Bcl-2 ratio and caspase activation. In vivo results, using a nude mouse xenograft model with HepG2 cell implants, demonstrated that administering aspirin at a dose of 100 mg/kg/day significantly reduced tumor growth compared to controls.

Additionally, other studies demonstrated that the COX-2 enzyme is overexpressed in inflammatory cancers and HCC. The COX-2 enzyme helps activate the profibrotic and proliferative signaling cascades including NF-κB pathways, protein kinase 3, among others [24,25,26]. By blocking COX-2 expression, aspirin helps reduce the effect of this pro-inflammatory pathway [27].

## 4. Aspirin’s Role in Management of Liver Disease

### 4.1. Aspirin in Metabolic-Associated Steatotic Liver Disease

Given that aspirin reduces levels of pro-inflammatory cytokines and prostaglandins, its effects were investigated in the context of MASLD, where inflammation levels are elevated (Table 1). A double-blind, randomized controlled trial by Simon et al. explored the effects of aspirin on 80 participants with documented steatotic liver disease either by histology or imaging [28]. Individuals with a history of alternative causes of liver disease, significant alcohol use, cirrhosis, or recent use of aspirin, antiplatelet, or anticoagulant medications were excluded from the study. The participants were randomized to aspirin 81 mg (n = 40) versus placebo (n = 40) for a total duration of 6 months. After 6 months, results showed that the absolute hepatic fat fraction was reduced by 10.3% (*p* = 0.009, with *p* < 0.05 considered statistically significant) in the aspirin group compared with the placebo [28]. Additionally, 42.5% of the aspirin group achieved a 30-percentage point reduction in hepatic fat compared to 12.5% with the placebo (*p* = 0.006). This was also evidenced by the magnetic resonance imaging proton density fat fraction (MRI-PDFF), which showed a change in liver fat content by 2.7% in the aspirin group versus 0.9% in the placebo (*p* = 0.004). This is promising as the reduction in hepatic fat content is a crucial treatment for MASLD and prevents disease progression [29,30,31]. The aspirin group also portrayed reduced fibrosis by vibration-controlled transient elastography (VCTE) of −1.1 kilopascal (kPA) compared to 1.7 kPA in the placebo group with a difference of −2.8 kPA [95% confidence interval (CI), −4.0 to −1.5]; *p* ≤ 0.001) [28].

Regarding adverse reactions, none of the participants developed anemia, thrombocytopenia, or life-threatening bleeding. However, the treatment duration was short, and most studies report a slightly elevated bleeding risk of approximately 1.7 events per 1000 person–years associated with low-dose aspirin use. It is also important to note that the small sample size and the fact that the clinical trial was conducted at a single center limits its generalizability. Furthermore, the study primarily focused on hepatic steatosis and did not investigate outcomes such as progression to cirrhosis or mortality [28].

Another population-based cohort study by Vell et al. investigated the impact of regular aspirin use on liver disease, with a focus on the development of MASLD and HCC. The study analyzed data from 460,755 participants drawn from two major biobanks—the UK Biobank and the Penn Medicine Biobank—who were followed for an average of 11.84 ± 2.01 years [32]. The primary outcome of this study was the diagnosis of new liver disease, identified using the “International Classification of Diseases and Related Health Problems” (ICD-10) coding system. Medication data were retrieved from the electronic health registry. Results showed that patients who regularly took aspirin experienced an 11.2% reduction in the risk of developing new liver disease over an 11.84-year follow-up period (hazard ratio [HR] = 0.888, 95% CI = 0.819–0.963; *p* = 4.1 × 10^−3^). Among the liver diseases, the code K76, which indicated MASLD, was significantly associated with regular aspirin use (HR = 0.882, 95% CI = 0.803–0.968; *p* = 8.0 × 10^−3^). This finding was further supported by liver MRI data, which showed that aspirin users had a lower risk of steatosis (>5% liver fat) compared to non-users (HR = 0.911, 95% CI = 0.843–0.985; *p* = 2.0 × 10^−2^).

The Penn Medicine Biobank cohort provided detailed insights into the duration of aspirin use, demonstrating a duration-dependent effect. The greatest reduction in liver disease risk was observed after at least one year of aspirin use (HR = 0.569, 95% CI = 0.425–0.762; *p* = 1.6 × 10^−4^). These effects were not observed with other medications, such as antiplatelets or nonsteroidal anti-inflammatory drugs (NSAIDs). This was attributed to aspirin’s unique mechanism of action as a potent, irreversible platelet inhibitor. Notably, the benefits of aspirin use were seen exclusively in men, with no significant effects in women. This suggests that the protective effects of aspirin may be influenced by gender differences [32].

Regarding side effects, regular aspirin users did not exhibit an increased risk of GI bleeding compared to non-users (HR = 0.904, 95% CI = 0.843–0.970; *p* = 4.7 × 10^−3^). Additionally, there was no increased risk of ulcer development (HR = 0.812, 95% CI = 0.788–0.836; *p* = 9.9 × 10^−300^). This absence of increased risk may be attributed to the fact that patients with a higher risk of bleeding were not prescribed aspirin thereby lowering the likelihood of aspirin-related complications [32]. Although this study demonstrated a significant impact of aspirin use on MASLD, the UK Biobank dataset lacks precise information on drug dosage and intake duration. Additionally, alcohol use was self-reported, which may have impacted the reliability of the data. Finally, reliance on ICD diagnoses could have resulted in missed patient groups or incorrect classification due to the absence of timely diagnoses.

Another prospective cohort study investigated the impact of daily aspirin use on 361 patients with biopsy-confirmed MASLD who were re-evaluated every 3–12 months until 9 years total duration (2006 until 2015) [33]. A total of 151 participants reported that they were on daily aspirin compared to 210 non-aspirin users. The primary outcome was to assess the risk of developing advanced fibrosis, measured using several indices including the Fibrosis-4 (FIB-4) score, NAFLD fibrosis score, and AST to Platelet Ratio Index (APRI). The study found that daily aspirin use was associated with a reduced progression to MASH (adjusted odds ratio [aOR] = 0.68, 95% CI = 0.37–0.89) and advanced fibrosis (aHR = 0.63, 95% CI = 0.43–0.85) in both genders. Additionally, a duration-dependent effect was observed, with significant reductions in fibrosis after 2 years of aspirin use and the most substantial benefits seen with at least 4 years of use (aHR = 0.50, 95% CI = 0.35–0.73). No similar benefits were observed with NSAID use. The study’s strengths include its detailed medication use data, long-term follow-up, and the availability of liver histology, which collectively enhance the robustness of the findings [33]. However, the study is limited by its reliance on self-reported aspirin use, which introduces the potential for recall bias and exposure misclassification. Additionally, it predominantly focuses on white individuals, limiting its generalizability to more diverse populations. The study also lacks data on other aspirin use patterns, adverse events, and the use of non-aspirin NSAIDs.

### 4.2. Aspirin and Hepatocellular Carcinoma

Aspirin was shown to have protective effects against HCC (Table 2), particularly among patients with CLD due to hepatitis B or C. Since hepatitis is one of the key risk factors for the development of HCC due to ongoing inflammation, studies targeted these populations to evaluate the effects of aspirin. A study by Jang et al. explored the effects of aspirin on HCC, liver-associated death, and major risk of bleeding in patients diagnosed with chronic hepatitis B (HBV), with and without cirrhosis [34]. Using the Korean database registry from 2002 to 2017, a total of 38,006 patients diagnosed with HBV were included in the study. The treated group (n = 19,003) was defined as patients who received aspirin < 100 mg for ≥90 consecutive days and was compared to the untreated group (n = 19,003) who were never prescribed aspirin. Propensity score matching was employed to minimize potential differences in covariates between the two groups. Patients diagnosed with hepatitis C (HCV), human immunodeficiency virus (HIV) coinfection, any cancer, a history of major bleeding, or those who received any antiplatelet therapy other than aspirin were excluded from the study. The patients were followed for a median of 6.7 years.

The findings revealed that, in patients without cirrhosis, aspirin use reduced the risk of HCC development compared to the non-aspirin group (aSHR, 0.87; 95% CI, 0.79–0.95; *p* = 0.002). Among patients with cirrhosis, no significant differences were observed between the two groups in terms of HCC (aSHR, 1.00; 95% CI, 0.85–1.18; *p* = 0.99), bleeding (aSHR, 1.05; 95% CI, 0.84–1.31; *p* = 0.68), or liver-related mortality (aSHR, 0.91; 95% CI, 0.72–1.14; *p* = 0.40) [30]. However, this study was conducted in a single Asian country, and the findings may not be generalizable to other racial groups. Additionally, data on HBV viral load and hepatitis B e antigen (HBeAg) status, which are important factors in HCC development and progression, were unavailable. As an observational study, it also did not explore the association between the duration of aspirin use and clinical outcomes [34].

Another study by Simon et al. also yielded positive findings in patients with hepatitis B or C on the incidence of HCC. The study analyzed 50,275 patients from 2005 to 2015 using the nationwide Swedish register to explore the effect of aspirin on HCC and liver-related mortality. Of the patients, 14,205 were taking low-dose aspirin (75–160 mg) for at least 180 days following their HCV or HBV diagnosis and were compared to non-aspirin users (n = 36,070). Findings revealed that the incidence of HCC was significantly lower in the aspirin group (4.0%) compared to the non-aspirin group (8.3%) (adjusted hazard ratio [aHR] = 0.69; 95% CI = 0.62–0.76). The risk reduction was also duration-dependent, with longer aspirin use associated with a greater decrease in HCC risk. Additionally, ten-year liver-related mortality was 11.0% among aspirin users, compared to 17.9% in non-users (aHR = 0.73; 95% CI = 0.67–0.81). Importantly, there was no significant increase in the 10-year risk of GI bleeding between the groups (risk difference = 0.9 percentage points; 95% CI = −0.6 to 2.4) [6]. As is consistent with the previous study, this study also lacked diversity in its patient population, which predominantly consisted of white individuals. Additionally, the study did not include data on actual adherence. These limitations emphasize the need for future research to explore the optimal timing for aspirin initiation, the minimum required duration, and the durability of its effects across diverse populations.

Another large cohort study by Lee et al. used Taiwan’s national database to explore the effect of aspirin on preventing HCC in patients diagnosed with an HCV infection from 1997 to 2011 [35]. The study excluded patients with HBV, HIV, and alcohol-related liver disease. It involved 2478 patients who continuously received aspirin (90 days or more) in comparison to 4956 patients who never received antiplatelet therapy [35]. Matching of the patients, 1:2, was carried out using propensity scores based on the index date and baseline characteristics. Results showed that the incidence of HCC among the aspirin group was significantly lower than the non-aspirin group (4.67%; 95% CI = 3.74–5.59% vs. 7.32%; 95% CI = 6.33–8.30%; *p* ≤ 0.001) [35]. A multivariable regression analysis showed that aspirin was independently associated with reduced HCC after adjusting for other factors such as age, gender, cirrhosis, statin use, and interferon therapy. This study is limited by its patient population and the presence of potential confounders between the two study groups.

Other studies were conducted to explore the difference between aspirin and non-aspirin medications (such as NSAIDs and antiplatelet agents) on HCC. A study by Tan et al. explored the effects of aspirin, NSAIDs, and antiplatelet agents on the incidence and recurrence of HCC [36]. Key findings of the study concluded that aspirin significantly lowered the risk of HCC incidence (HR = 0.51, 95% CI = 0.36–0.72) and improved liver-related mortality (odd ratio [OR] = 0.32, 95% CI = 0.15–0.70) with a small increased risk of GI bleeding (OR = 1.32, 95% CI = 1.08–1.94). The study also showed that the risk of HCC recurrence was significantly reduced in patients who received NSAIDs and antiplatelets (HR = 0.73, 95% CI = 0.63–0.84) [36].

Similar findings were seen in a prospective study by Sahasrabuddhe et al. where they examined the impact of aspirin compared to other NSAIDs on the progression of liver disease to HCC using the National Institute of Health-AARP Diet and Health study cohort. This study included 300,504 participants aged 50 to 71 years. A risk factor questionnaire was administered to assess aspirin use over the past 12 months, including its frequency. In total, 219,291 (73%) were taking aspirin, while 168,499 (56.1%) used non-aspirin NSAIDs [37]. The study found that aspirin use was associated with a reduced risk of developing HCC (relative risk [RR] = 0.59; 95%, CI = 0.45 to 0.77) and a decreased risk of death related to CLD (RR = 0.55; 95% CI = 0.45 to 0.67) compared to non-users. In contrast, non-aspirin NSAIDs were linked to a reduced risk of death from CLD (RR = 0.66; 95% CI = 0.48 to 0.91) but did not show a significant reduction in the risk of HCC (RR = 0.96; 95% CI = 0.63 to 1.47) [37]. The study concluded that aspirin appears to offer better liver protection than other non-aspirin NSAIDs. However, the study had limitations, including the lack of consideration for major risk factors such as hepatitis B or C, insufficient data on NSAID dosage and strength, and reliance on self-reported data, which may introduce biases. Additionally, the risk of GI bleeding was not addressed, highlighting the need for further research to balance the benefits of aspirin against potential risks [37].

**Table 2:** Aspirin in HCC. This table summarizes the effect of aspirin on hepatocellular carcinoma.

Study	Design	Population	Primary Outcome	Other Outcomes	Adverse Effects
Jang et al. (2022) [34]	Retrospective cohort	329,635 adults with HBV; 20,200 on aspirin vs. 309,435 without antiplatelet therapy.	10-year HCC incidence: 9.5% aspirin users vs. 11.3% non-users (aSHR 0.85).	Lower liver-related mortality (aSHR 0.80).	No significant increase in major bleeding.
Tan et al. (2021) [36]	Systematic review and meta-analysis	19 studies; 147,283 with CLD (HBV, HCV) on NSAIDs/APT.	Reduced HCC risk (HR 0.51); improved liver-related mortality (OR 0.32).	Reduced HCC recurrence with NSAIDs (HR 0.80).	Slightly increased GI bleeding.
Simon et al. (2020) [6]	Retrospective cohort	50,275 HBV/HCV adults; 14,205 on low-dose aspirin (≤160 mg) for ≥90 days.	Lower HCC risk (HR 0.69); reduced liver-related mortality (HR 0.73).	—	No significant difference in GI bleeding.
Lee et al. (2020) [35]	Randomized, double-blind trial	14,205 HBV patients on low-dose aspirin (≤160 mg) vs. placebo.	10-year HCC incidence: 4.0% aspirin users vs. 8.3% non-users (aHR 0.69).	Lower liver-related mortality (aHR 0.73).	No significant GI bleeding difference.
Sahasrabuddhe et al. (2012) [37]	Prospective cohort	14,205 aspirin users (low-dose, ≤160 mg).	Reduced HCC risk (RR 0.59).	Lower mortality from CLD (RR 0.55); non-aspirin NSAIDs reduced CLD mortality (RR 0.74).	—

Abbreviations: HBV = chronic hepatitis B; HCV = chronic hepatitis C, HCC = hepatocellular carcinoma; aSHR = adjusted sub-hazard ratio; HR = hazard ratio; OR = odds ratio; RR = relative risk; CLD = chronic liver disease; GI = gastrointestinal; NSAIDs = nonsteroidal anti-inflammatory drugs; APT = antiplatelet therapy.

## 5. Safety and Risks

Cirrhotic patients are at an increased risk of bleeding due to multiple factors affecting their coagulation system and platelet function. Thrombocytopenia, commonly seen in these patients, arises from both decreased thrombopoietin levels and heightened splenic sequestration [38,39]. Moreover, impaired liver function results in reduced synthesis of essential coagulation factors, leading to a prolonged international normalized ratio (INR) [40]. Elevated portal venous pressure further compounds the bleeding risk by contributing to the development of varices, which are vulnerable to rupture and can lead to significant hemorrhage. According to the American Association for the Study of Liver Diseases (AASLD), portal hypertension itself is an independent risk factor for procedure-related bleeding [40]. These combined mechanisms make cirrhotic patients more susceptible to bleeding than those without cirrhosis.

Although aspirin was shown to reduce the risk of liver-related complications such as fibrosis progression, HCC, and overall mortality, there remains a valid concern about its potential to cause significant bleeding, particularly in high-risk patients and those with cirrhosis. A systematic review and meta-analysis by Zheng et al. examined the benefits and risks of aspirin use for the primary prevention of cardiovascular disease, analyzing data from 13 randomized clinical trials involving a total of 164,225 participants [41]. It showed that aspirin use is associated with a significant reduction in cardiovascular mortality (HR for composite cardiovascular outcome = 0.89; CI = 0.84–0.95); however, it was counterbalanced by the substantial increase in major bleeding events (HR = 1.43; CI = 1.3–1.56). The types of bleeding events included GI bleeding and intracranial hemorrhage, leading to a significant increase in mortality [41]. These findings highlight that while aspirin can modestly reduce cardiovascular complications, it does so at the expense of an increased risk of major bleeding.

The ASPREE randomized controlled trial investigated the effects of low-dose aspirin (100 mg) in elderly patients (aged ≥ 70 years, or ≥65 years for U.S. minority groups). The trial included 19,114 participants with a median follow-up of 4.7 years, of whom 9525 were assigned to receive aspirin and 9589 to receive a placebo. Results indicated a significantly increased risk of GI bleeding, with upper GI bleeding (HR = 1.87; 95% CI = 1.32–2.66) and lower GI bleeding (HR = 1.36; 95% CI = 0.96–1.94), suggesting an overall 60% increase in GI bleeding in this population. A multivariate analysis further revealed that additional risk factors, such as smoking, hypertension, chronic kidney disease, and obesity, heightened the bleeding risk. The 5-year risk of bleeding was noted to be 0.25% in a healthy older adult, compared to 5.03% in an older adult with additional risk factors [42]. Therefore, according to the latest guidelines from the American Heart Association (AHA), aspirin is not recommended for primary prevention in adults over 70 years old or in those with an increased risk of bleeding [43]. This is particularly important as patients with liver disease are at an increased risk of bleeding.

This aligns with another meta-analysis of four studies, which revealed that aspirin users have a 32% higher risk of developing GI bleeding compared to non-aspirin users (HR: 1.32; 95% CI: 1.08–1.94). The risk was even higher, increasing twofold, in those on dual antiplatelet therapy (HR: 1.32; 95% CI: 1.08–1.94). Similarly, a retrospective study by VW et al. demonstrated a dose-dependent risk of GI bleeding among aspirin users, with patients taking aspirin for ≤2 years having a higher risk of GI bleeding (HR: 1.73; 95% CI: 1.07–2.79) compared to non-users. However, this risk decreased after 5 years (HR: 0.79; 95% CI: 0.19–3.21) [44].

Although there is an increased risk of bleeding, certain strategies can be employed to mitigate these risks. According to the American College of Gastroenterology, the use of proton pump inhibitors was found to significantly reduce the incidence of upper GI bleeding in patients taking low-dose aspirin [45]. Additionally, optimizing other comorbid conditions, such as chronic kidney disease and hypertension, is crucial in reducing bleeding complications. Therefore, the AASLD guidelines suggest that aspirin can be used in patients with liver disease, including cirrhosis, but with careful monitoring and consideration of bleeding risk [40,46]. Regular and close monitoring of liver function tests, coagulation parameters, and assessments for bleeding may aid in the early detection and management of bleeding complications [40].

## 6. Level of Evidence and Future Research

Based on the most recent meta-analysis, which investigated seven different studies with a total population of 51,799 participants, the association between aspirin use and HCC risk was found to have a pooled HR of 0.51 (95% CI, 0.36–0.72). In patients diagnosed with HBV or HCV, aspirin was shown to be particularly beneficial, with studies indicating a duration-dependent effect—longer use (≥3 years) is associated with a more significant reduction in risk [34,35]. However, due to clinical heterogeneity among the participants, including variations in aspirin dose, duration, and follow-up period, the current evidence is considered moderate according to GRADE certainty [36,47]. Although current data suggest that aspirin does not significantly increase bleeding risks, patients with liver disease should be carefully monitored due to their heightened susceptibility.

Further research is needed to fully explore the molecular pathways involved as the exact protective mechanism of aspirin remains unclear [6]. Additionally, the optimal dosage and duration of aspirin therapy for liver disease prevention have not been well defined. While some studies have shown a dose-dependent reduction in HCC risk [28], standardized guidelines are lacking. Larger randomized trials are needed to fully establish the long-term safety and efficacy of aspirin in liver disease management, as well as to explore the influence of factors such as gender and duration of use. The safety profile of aspirin, particularly in patients with different liver diseases, needs to be better characterized. Most of the current evidence is derived from observational studies; therefore, larger scale randomized controlled trials are necessary to assess and confirm the benefits versus risks of using aspirin in liver disease populations. Lastly, other medications, including statins, ursodeoxycholic acid, probiotics, vitamin E, and branched-chain amino acids, were also investigated for managing CLD. Future research could focus on comparing these therapies or exploring their potential benefits as adjuncts to aspirin treatment to better understand and consolidate their therapeutic roles [4].

## 7. Conclusions

Aspirin has shown significant benefits in the management of liver disease. Numerous studies have demonstrated its ability to reduce hepatic fat content, prevent fibrosis progression, and lower the risk of liver-related complications such as hepatocellular carcinoma (HCC) and mortality, particularly in patients with hepatitis B or C. The protective effects of aspirin, especially with prolonged use, highlight its potential role in preventing MASLD and HCC.

However, these benefits must be carefully weighed against the potential risks of bleeding, particularly in patients with cirrhosis or other factors that increase the likelihood of hemorrhagic complications. Currently, there is insufficient evidence to recommend a specific dose or duration for its use. Balancing between aspirin’s therapeutic effects and its bleeding risks will be essential in defining its role as a preventive and therapeutic agent in liver disease. Further research is needed to comprehensively evaluate aspirin’s potential and to explore its integration into treatment strategies for liver disease.

## Figures and Tables

**Table 1:** Aspirin in MASLD. This table summarizes the different studies analyzing the use of aspirin in metabolic-associated steatotic liver disease.

Study	Design	Population	Primary Outcome	Other Outcomes	Adverse Effects
Simon et al. (2024) [28]	Double-blind, randomized controlled trial	80 participants with MASLD (40 aspirin, 40 placebo)	10.3% reduction in hepatic fat fraction in aspirin group compared to placebo (*p* = 0.009).	42.5% of aspirin group achieved ≥30% fat reduction vs. 12.5% in placebo (*p* = 0.006).	No anemia, thrombocytopenia, or life-threatening bleeding.
Vell et al. (2023) [32]	Population-based cohort study	460,755 participants from UK Biobank and Penn Medicine Biobank	11.2% reduction in new liver disease with aspirin use (HR = 0.888; *p* = 4.1 × 10^−3^).	Lower MASLD risk (HR = 0.882; *p* = 8.0 × 10^−3^); duration-dependent effects in men only.	No increased risk of GI bleeding or ulcers.
Simon et al. (2019) [33]	Prospective cohort study	361 MASLD patients (151 on daily aspirin)	Reduced progression to advanced fibrosis (aHR = 0.63; *p* < 0.05).	Duration-dependent effect: significant fibrosis reduction after 2–4 years of use.	—
